# Easy DNA Modeling and More with GraphiteLifeExplorer

**DOI:** 10.1371/journal.pone.0053609

**Published:** 2013-01-07

**Authors:** Samuel Hornus, Bruno Lévy, Damien Larivière, Eric Fourmentin

**Affiliations:** 1 Equipe ALICE, Inria Nancy - Grand Est, Villers-lès-Nancy, France; 2 Fourmentin-Guilbert Scientific Foundation, Noisy-Le-Grand, France; Semmelweis University, Hungary

## Abstract

The GraphiteLifeExplorer tool enables biologists to reconstruct 3D cellular complexes built from proteins and DNA molecules. Models of DNA molecules can be drawn in an intuitive way and assembled to proteins or others globular structures. Real time navigation and immersion offer a unique view to the reconstructed biological machinery.

## Introduction

Thanks to structural biology and *omics* technologies, biologists have now collected large amounts of data regarding *individual* biological molecules. In order to build a system view of living organisms, new engineering tools like large scale 3D modeling software are required.

The GraphiteLifeExplorer 3D modeling tool is being developed with the ultimate goal to reconstruct a complete bacterial cell from its individual parts. In addition to the 3D reconstruction of protein arrangements, one of its main features is the ability to model, in an intuitive manner, any DNA molecules of arbitrary length and shape at the resolution of one base pair. It is intended to help biologists to formulate and test hypothesis on some unavailable information such as the local and global folding of the DNA, the possible positions of proteins relative to the DNA, etc.

This paper describes the process and the tool to achieve 3D modeling of cellular complexes with the ability to add DNA. The program architecture is then presented as well as technical details about the DNA representation.

## The 3D Modeling Process

**Figure 1:**
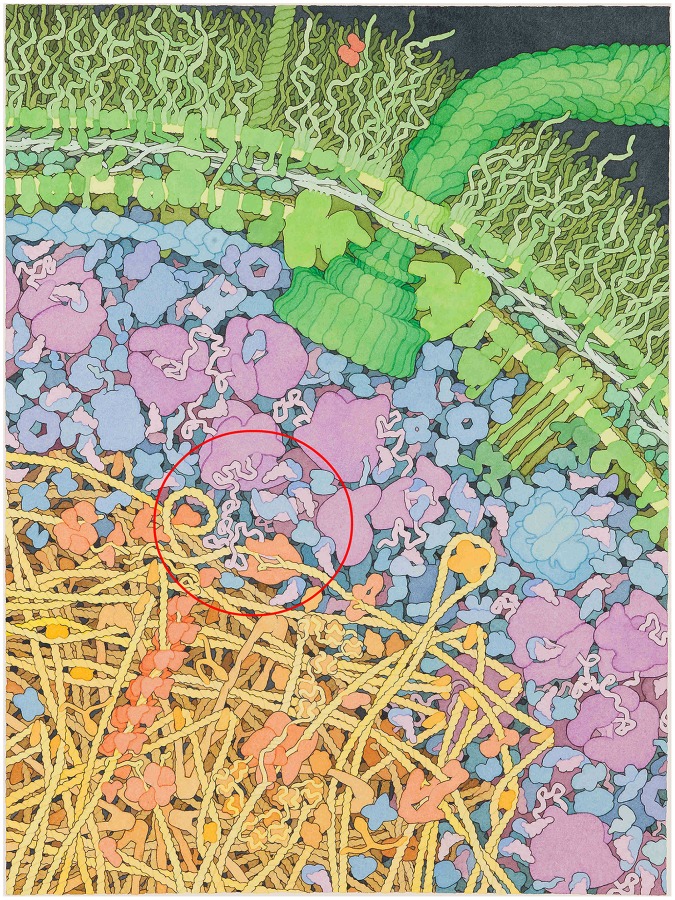
Realistic illustration of the interior of an *E. coli* bacterial cell by D.S. Goodsell (TSRI). It shows the spatial organization of the cell and its crowding: 1/The nucleoid (in yellow/orange) is made of DNA containing the genetic information and of proteins interacting with the DNA (to repair it for instance) 2/Some of these proteins read the genetic information and transfer it (see the red circle) into the cytoplasm (in blue/purple) where it serves to build new proteins performing most of the tasks inside the cell 3/Some of these proteins go back to the nucleoid, others remains in the cytoplasm, still others go to the membrane (in green). Other elements of this organization are the cytoskeletons (the blue filament in the upper left corner of the illustration), long and filamenteous “molecular motorways” that track various proteins from one pole of the cell to the other one. About genome organization in *E. coli*, the DNA is condensed into a compact structure called the nucleoid and is organized into macrodomains [25]. However the fine spatial organization of the DNA inside a macrodomain is still largely debated. This illustration is an original photograph of David Goodsell's painting. It differs from the version published in [26]–[27]. With kind permission of David S. Goodsell.

**Figure 2:**
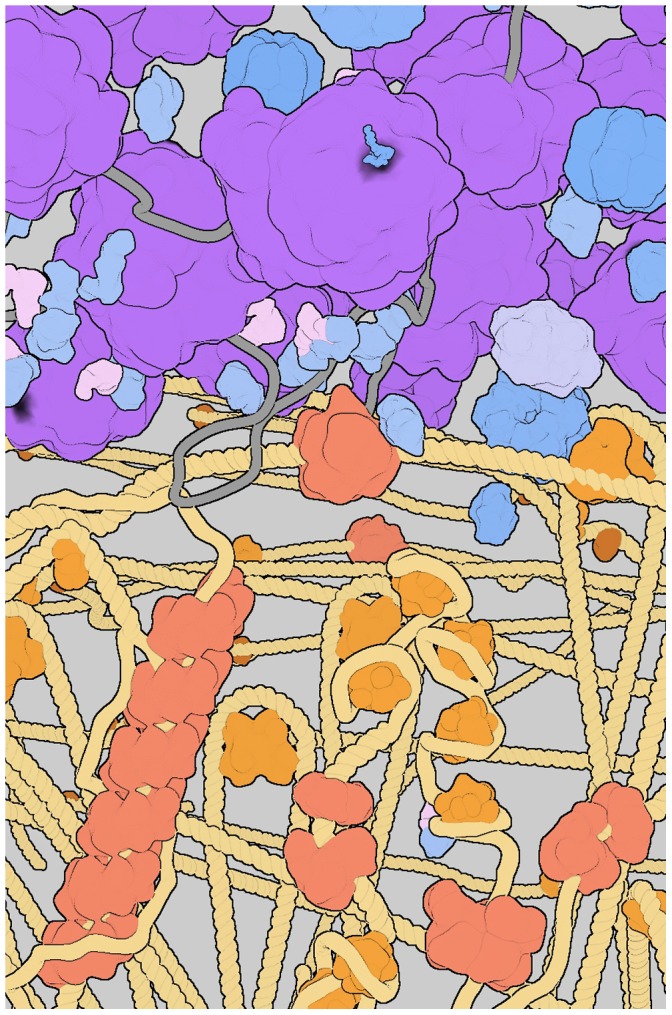
Partial reproduction with GraphiteLifeExplorer of the *E. coli* illustration shown **figure 1**. This multicomponent scene contains several ribosomes and proteasomes, many tRNA and elongation factors, various enzymes, a RNA polymerase transcribing the DNA, a replication fork, a RecA filament and two RecBCD complexes, several DNA binding proteins.

Bacterial cells are internally organized [1]–[3]. Figure 1 gives an illustration of the spatial organization for a bacterial cell. Figure 2 shows at a glance the potential of the GraphiteLifeExplorer tool to virtually rebuild a bacterium.

### A Global View

The modeling process carried out with GraphiteLifeExplorer is made of the following steps (see also [4]):

- The first step is the **collection of structural data** on the complex or the process of interest.
- The 3D shape of DNA molecules is generally unconstrained but can be generated using **geometry modeling**. The atomic structure of a protein can also be transformed into a surface representation that allows an immediate and intuitive understanding of its shape. Some regions can be distinguished with different colors to highlight residues involved in protein-protein interaction for instance.
- The next step is the manual assembly of the various components (**scene composition)**. Their position and orientation are known for instance from an electron density map after a more or less flexible fitting of the proteins within the envelope has been carried out [5] or from crosslinking data [6].
- Once the scene has been created, the biologist can **navigate in real-time** within the scene to explore it.
- The 3D scene can be exported in order to be used in external software (for physics-based animation for instance).

### Geometry Modeling with GraphiteLifeExplorer

**Figure 3:**
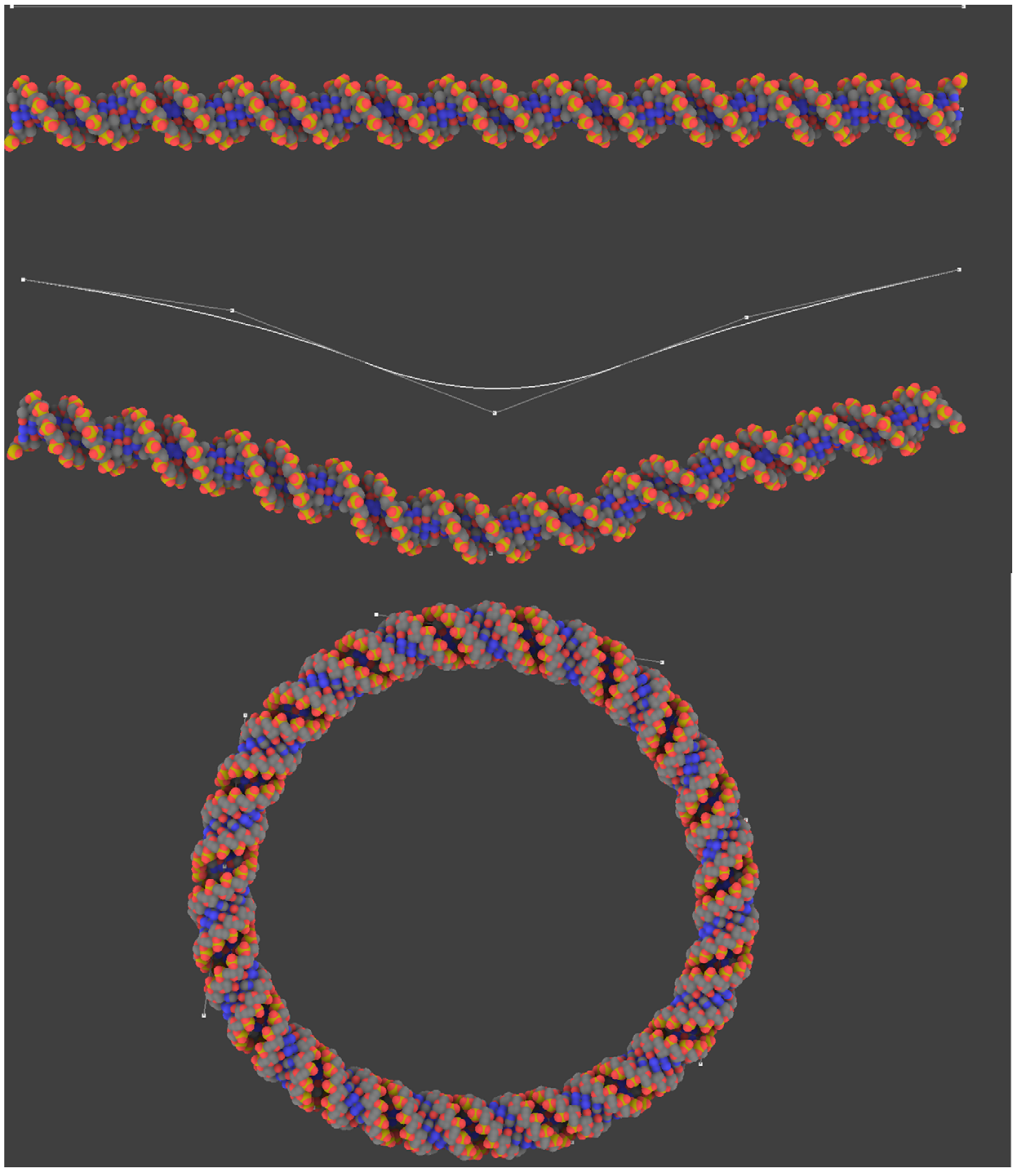
The DNA modeling process. To create DNA the user draws a path which can be adjusted in the three directions of space thanks to control points. The whole atomic structure is built upon the path. Closed structures can be created as shown by the circular A-DNA model.

**Figure 4:**
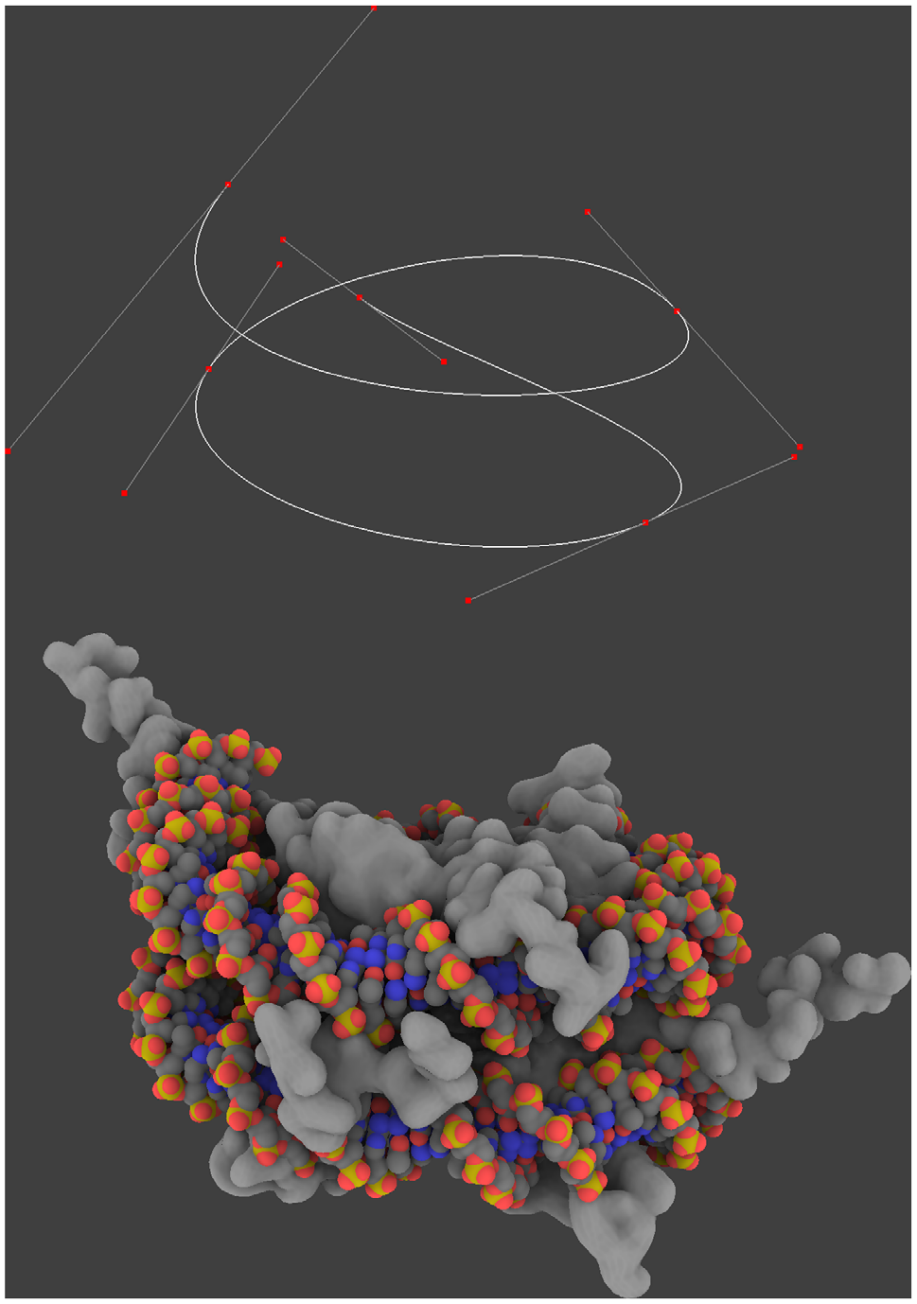
The DNA modeling process. GraphiteLifeExplorer is equipped with a tool allowing to draw complicated shapes (like wrapping of DNA around a nucleoparticle for example) quickly and easily with a few number of control points. Note that the twist of the DNA can be locally or globally adjusted allowing one particular atom of the DNA to face one particular residue at the surface of the protein without changing the whole conformation (see figure 5).

**Figure 5:**
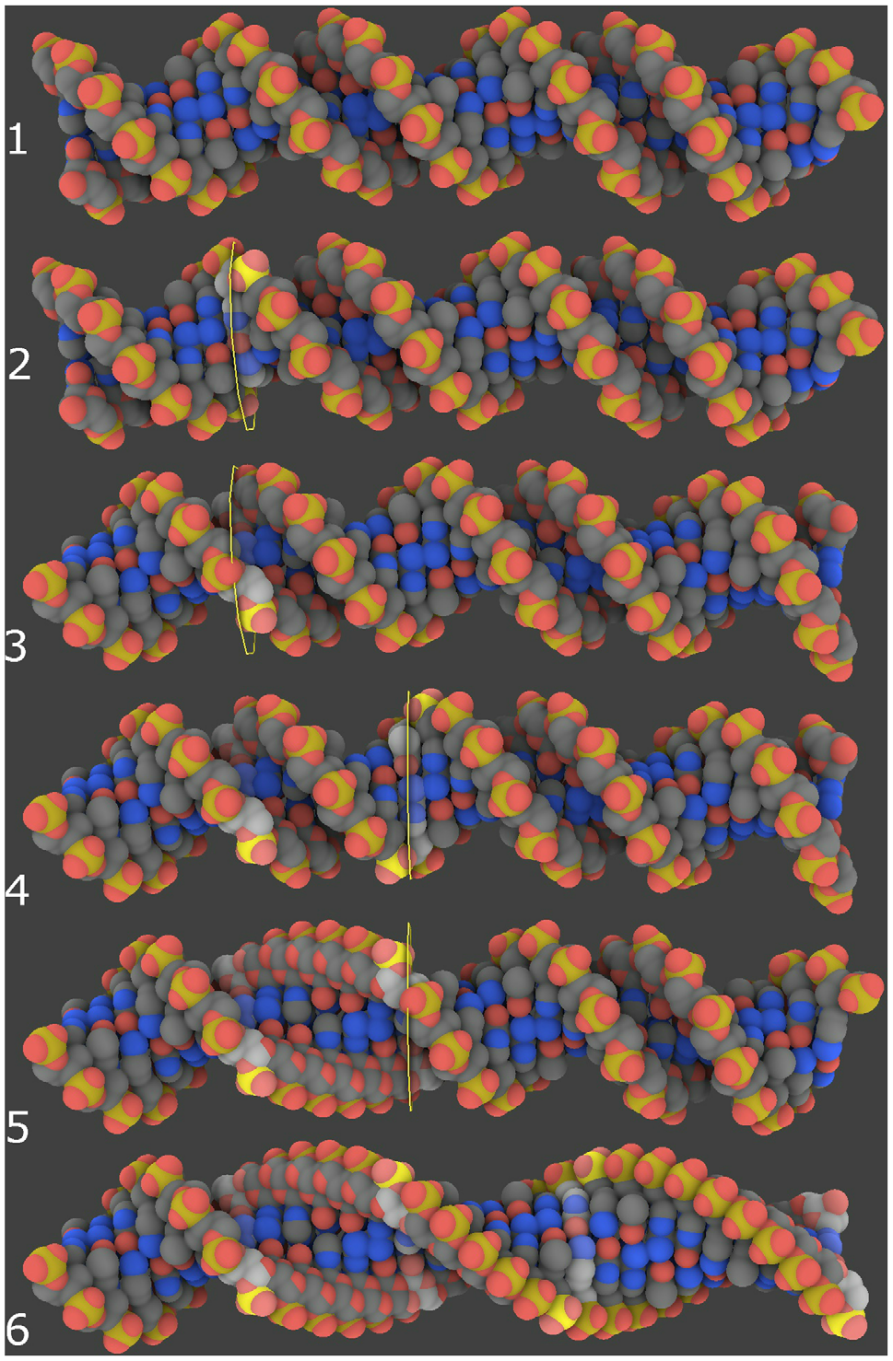
Twisting DNA. From top to bottom: 1/The initial DNA strand 2/A base pair is selected and 3/Twisted. All pairs follow the twist 4/Another pair is selected and 5/Twisted. The DNA is unwound between the two selected pairs 6/More pairs have been twisted.

Since no fixed structure is available, some macromolecules have to be modeled *de novo* (DNA, linkers, membrane…). Modeling DNA is intuitive and easy in GraphiteLifeExplorer as explained Figures 3 and 4. The DNA strand is built by modeling its helical axis as a curve in space. Currently, GraphiteLifeExplorer allows the edition of quadratic and cubic Bézier curves [7]. The Bézier curves are modifiable: their control points can be created, moved, deleted and duplicated to model DNA strands of arbitrarily complicated shape, open or closed. At any time during the interactive modeling session, the curve can be visualized using different representations: as a plain line, as a tube or as an atomic representation. The visualization can be partially transparent to allow editing the curve while seeing the DNA model move and transform in real-time. When the overall shape of the DNA has been fixed, it is possible to fine tune the position of its base pairs: the user can force the angular position (the twist) of any chosen base pair around the curve. This angular constraint is then automatically propagated to the neighboring pairs to get as close as possible to the canonical DNA helix. For example, DNA can be locally untwisted, as illustrated in Figure 5. Unwinding the helices can lead to chemically impossible structures without inappropriate molecular dynamics refinement. This functionality allows the user to find a DNA structure that is intermediate between the untwisted helices and the expected structure. By exporting the DNA structure to a PDB file format, it is possible to perform a further refinement step.

3DNA [8] or Nucleic Acid Builder (NAB) [9] are powerful programming environments dedicated to the chemically rigorous structural building of nucleic acids but cannot be used to create arbitrary DNA shapes in an interactive manner. A portion of DNA can effectively be generated in NAB over a cubic Bezier curve but the control points must be specified numerically since there is no interactive graphical user interface. 3DNA is also not interactive. However, the web-based interface to the program (Web 3DNA) [10] greatly helps the user model a DNA/protein complex. The chosen approach is to choose a PDB file representing the structure of the protein of interest, then to enter the sites onto the protein where the DNA binds and to launch a calculation yielding a DNA 3D structure wrapping the protein. GraphiteLifeExplorer's approach is faster (but less accurate) and gives the user more freedom (e.g. wrapping DNA around a protein plus adding a loop).

**Figure 6:**
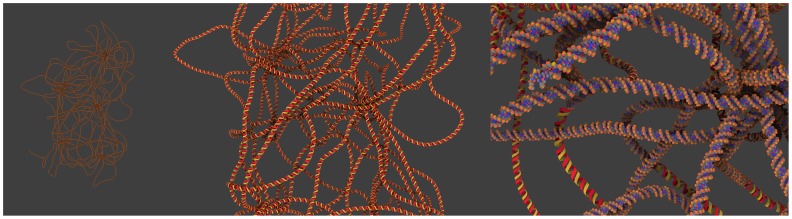
Visualization of DNA simulation results. The comprehension of the (dynamic) configuration adopted by the DNA, of its packing into the cell is one of the major goals of biology. Numerical simulations address the topology of longer and longer DNA structures like plasmids. Here is shown the compaction of 28,000 DNA base pairs resulting from the action of fifty transcription factors (data shown courtesy of Ivan Junier; see [28] for details about the self-avoiding worm-like chain numerical simulation). Left image: The DNA is displayed as a thick line. Middle image: The DNA is displayed as a helicoidal double ribbon when the user zooms in. Right image: A transition between the helicoidal double ribbon rendering and the atomic rendering occurs when the user brings the camera closer to the object.

Specific features: GraphiteLifeExplorer can also import coarse grained data (possibly obtained from simulation) as a sequence of 3D points and turn it into a smooth interpolating curve. The DNA molecule can then be explored at several Levels of Detail (LoD) (Figure 6).

**Figure 7:**
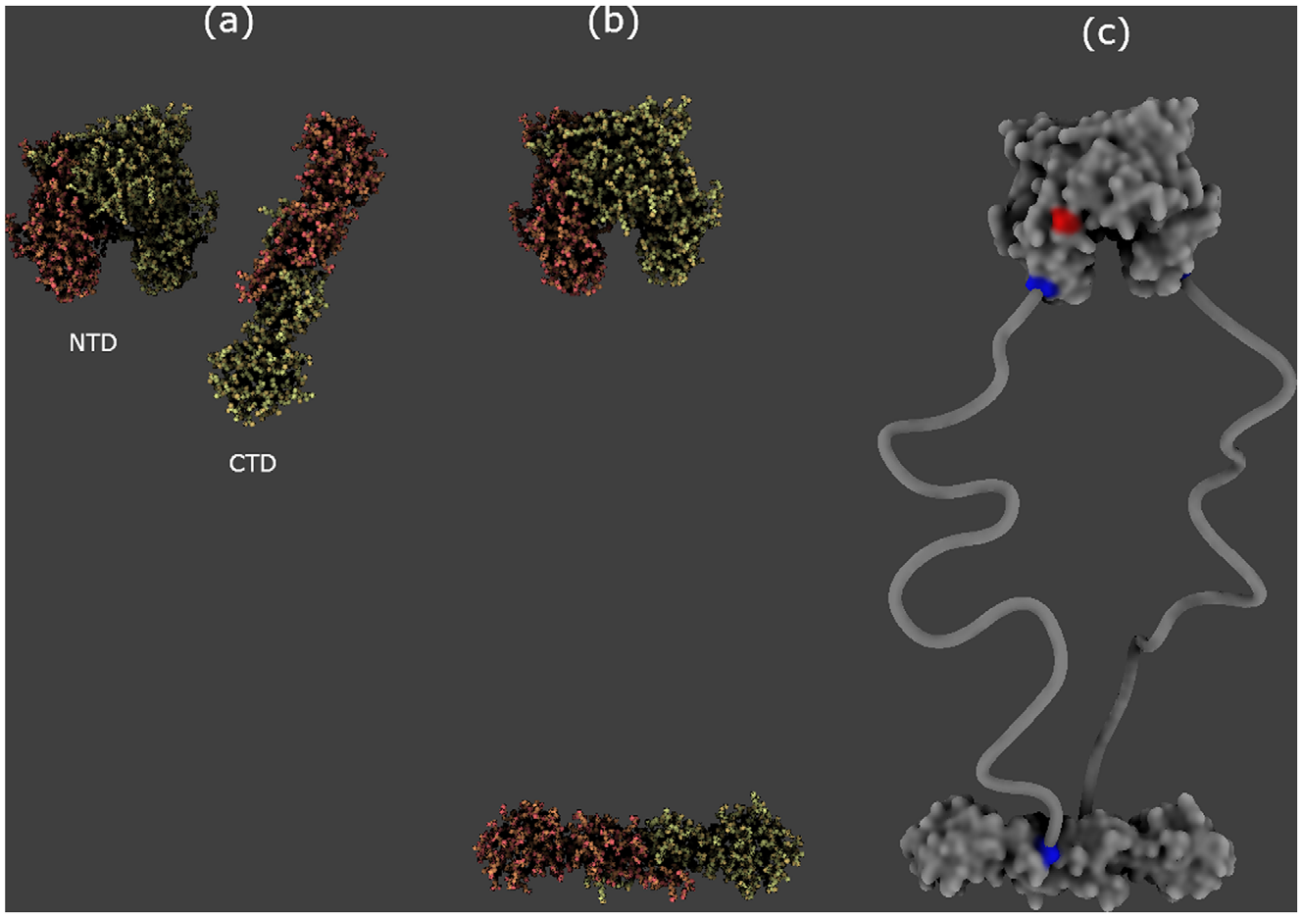
Reconstruction process of a complex in GraphiteLifeExplorer. Case of the bacterial DNA repair protein MutL: (a) the N-terminal (NTD) and the C-terminal domain (CTD) of MutL are imported under the form of a full-atom representation from their respective PDB file. (b) The CTD is moved manually relatively to the NTD. (c) A surface appearance is given where residues 331 of the NTD and 432 of the CTD are colored in blue to help the modeling of two 40 nm long amino-acid linkers connecting the two domains. Although their amino-acid sequence is known, the linkers are disordered and therefore missing in the attempts to experimentally solve the 3D structure of the whole MutL. Here, the linkers have been built as a simple tube of fixed length and in agreement with AFM images [29]. Some residues at the surface of the N-ter domain have been colored in red to show where another repair protein called MutH interacts with MutL. PDB code N-ter: 1B63, PDB code C-ter: 1x9z (both subunits have been reconformed as suggested in [30]).

A number of proteins exhibit large disordered domains under the form of linkers without any secondary structure. Figure 7 illustrates the modeling of a linker in GraphiteLifeExplorer. To date, a linker is represented under the form of a tube.

### Usefulness and Limitations: Shapes vs. Structure

By using simple geometric shapes for DNA, GraphiteLifeExplorer helps biologists to study and discriminate between the global shapes likely taken by DNA in contact with proteins. Information like protein-protein or DNA-protein crosslinks [11]–[12] or tomograms might provide further proofs of the model.

Powerful for studying shapes, GraphiteLifeExplorer is not designed for detailed local structure studies. Single/double nicks, mismatchs, kinks or interbase stretches cannot be created by using the tool exclusively. Moreover a few structural deficiencies can subsist such as O3’ – P bond abnormal stretches between adjacent residues of the same chain. Clashes between atoms can also occur at the apex for severe curvatures. The user needs to be aware that cyclized DNA can be created (figure 3) but closing of DNA whose length is not a multiple of 10.4 requires the pitch to slightly differ from 10.4 base pairs per turn. Refinement can be done by exporting the model in the Protein Data Bank (PDB) file format (see § Export capability) and by performing an Amber based-molecular dynamics [13].

**Figure 8:**
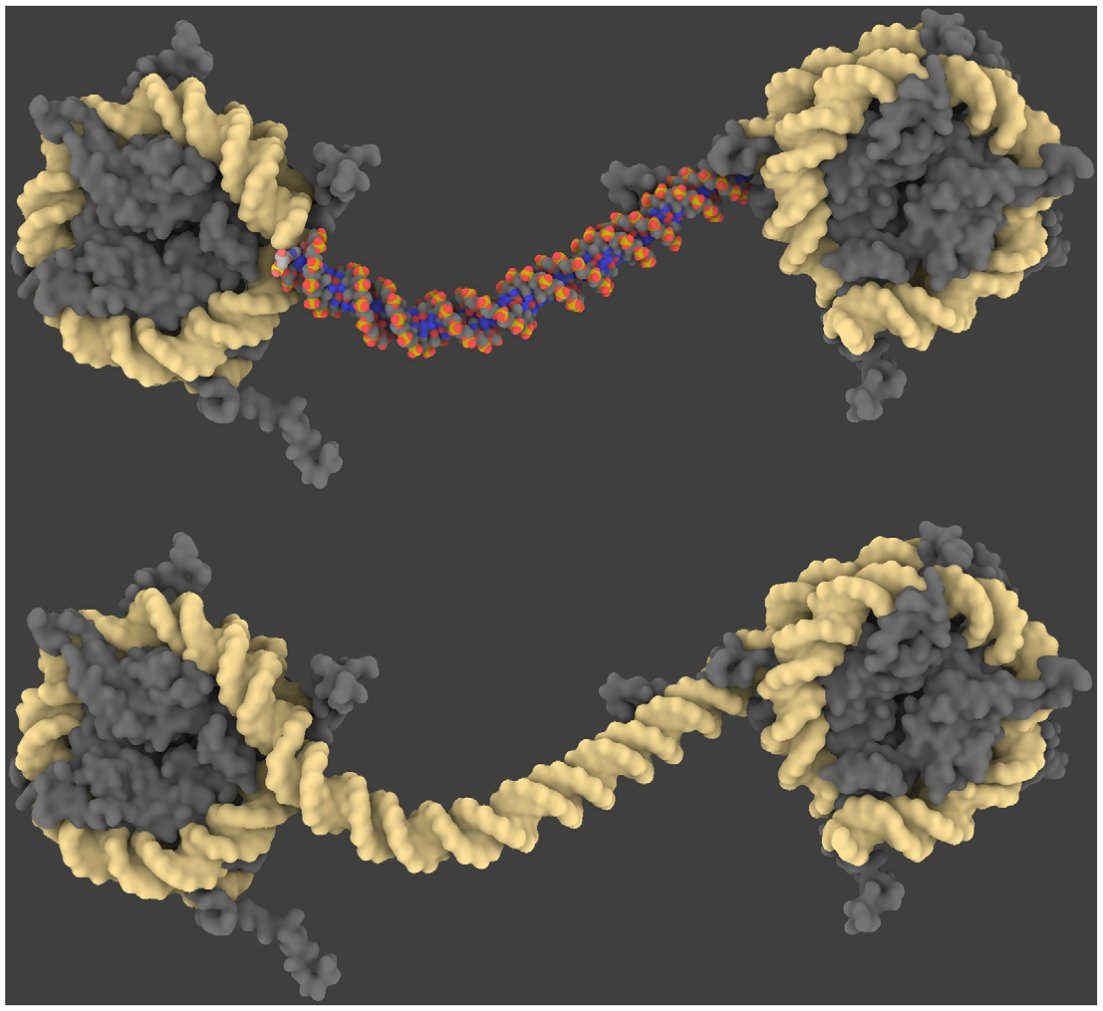
Connecting nucleic acid structures. Missing DNA between two nucleosomal complexes containing DNA (PDB code 1KX5) is modeled with GraphiteLifeExplorer (upper image). The “welding” between modeled DNA and DNA from crystallography is carried out in a simple manner with Web 3DNA [10]. Whole DNA is shown as an isosurface in GraphiteLifeExplorer (lower image). Molecular dynamics would then be necessary to take relaxation effects into account.

Associated with a tool like Web 3DNA [10] GraphiteLifeExplorer can nevertheless help the structural study of interaction with proteins. In the example of figure 8, GraphiteLifeExplorer is used to create the missing part between two nucleosomal particles. The connection with the experimental DNA structures is carried out with Web 3DNA. Obviously, molecular dynamics is mandatory at the end of the process as the connecting DNA portion must mechanically and structurally accommodate with the structural constraints on each side.

### Enhanced Real-time Navigation

**Figure 9:**
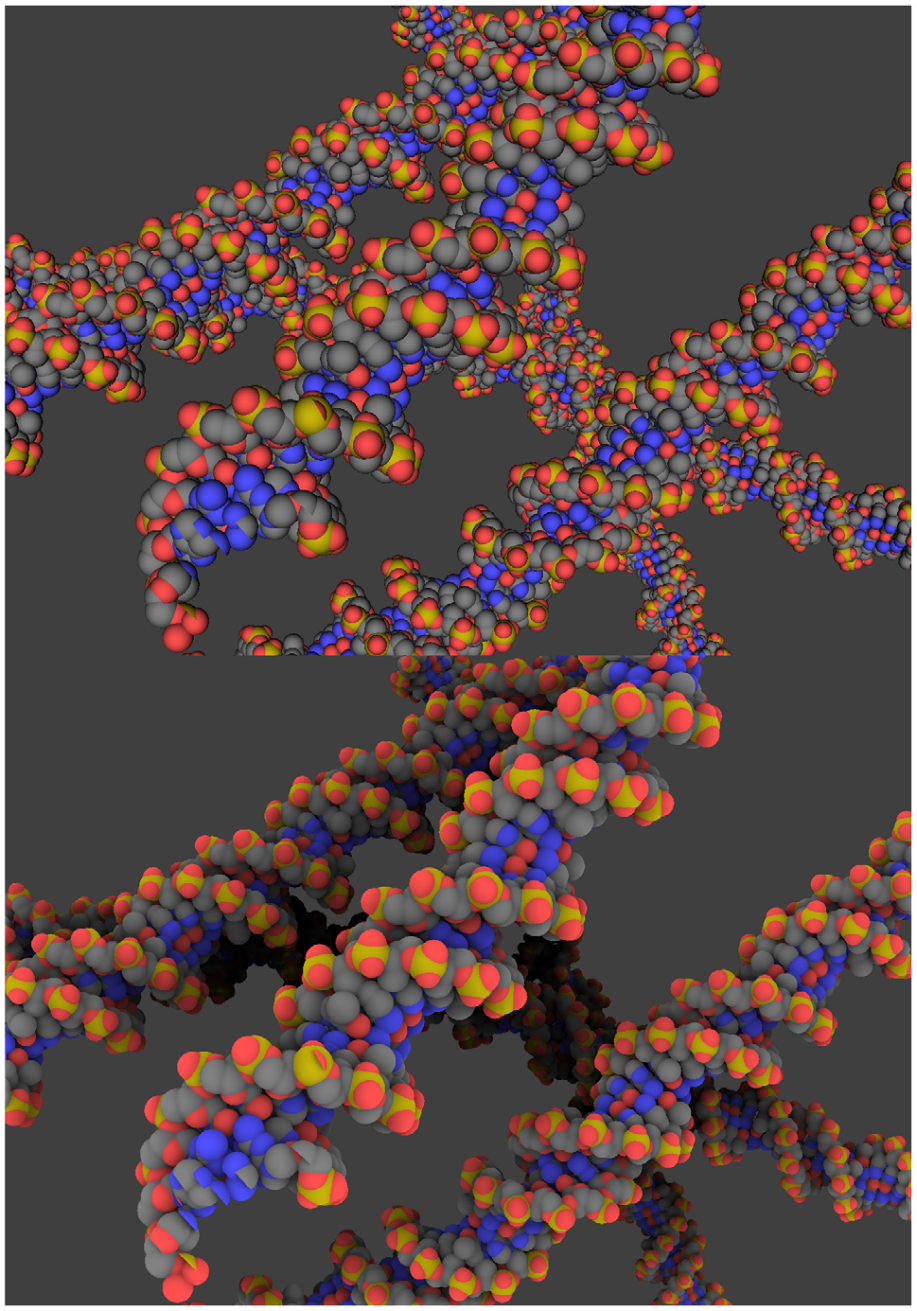
Sophisticated rendering. Top: Simple lighting from the viewpoint. Bottom: Ambient occlusion without lighting.

Real-time navigation (coupled with scene composition) is a valuable experience to inspire new questions. Rendering is enhanced in GraphiteLifeExplorer by real-time ambient occlusion, providing a better perception not only of the protein shape [14] but also of the relative position of the components in the scene (Figure 9). Various effects (like cartoon rendering, figure 2) can be used for illustration purpose.

### Export Capability

GraphiteLifeExplorer relies on its export capabilities coupled with well-established tools for physics-based animation or minimization computations. A scene can be exported either as a set of full atom representations in the Protein Data Bank (PDB) file format or as a set of geometric shapes.

#### PDB export

**Figure 10:**
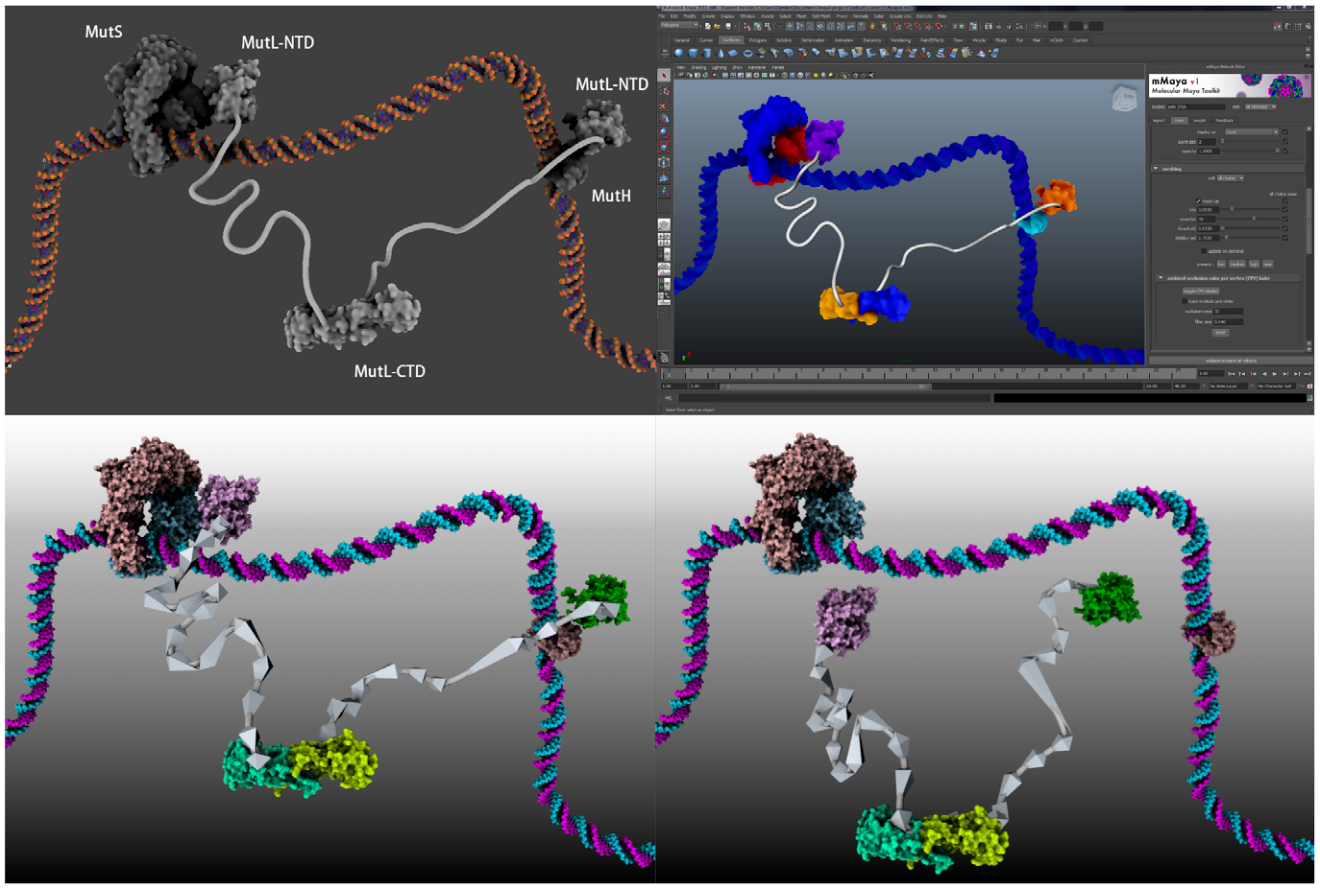
3D model export. Upper left image: This 3D scene has been made in GraphiteLifeExplorer and exported (upper right image) in Maya thanks to Molecular Maya (http://www.molecularmovies.com/toolkit/) and (lower images) in the Blender 3D tool thanks to the ePMV plugin [31]. In Blender, a script written by L. Autin (Scripps) superimposes an inverse kinematic armature to the linkers. This mechanical articulation greatly helps and facilitates the animation of the proteins from a first position (right figure) to a second position (left figure): thanks to the IK chain, moving the pink and green domains results in a reconformation of the linkers, pushed or pulled like a chain, in the deformation of the geometry (the tubes representing the linkers) attached to the linkers, and in the displacement of the CTD domain (green-blue/yellow). The model can serve as an interactive data-constrained thinking tool to help a lab contemplate plausible dynamics for this system. Note that the inverse kinematic joints of such an armature can be set at each alpha-carbon of any backbone if the linkers is no more a tube but an aminoacid based linker modeled with tools like Phyre2 [32] and be combined with physics solvers hosted in the high-end 3D packages.

Each component of a scene (DNA and proteins) can individually be saved in the PDB format in which new atomic coordinates account for the spatial transformation that occurred during the composition of the scene. Figure 10 shows an example of a scene initially made with GraphiteLifeExplorer and visualized in Maya and Blender. Exporting in PDB thus allows the user to perform any task requiring a full-atom description of the components in an external tool.

#### Geometry export

As shown Figure 10, tubular shaped linkers and molecular surfaces can be exported as geometric objects (surfaces) in a common 3D format called Virtual Reality Markup Language (VRML). Doing so, any connection to the atomic description is definitively lost. This geometric export is useful when the user wishes to explore the scene in another tool with no PDB import capability, the user does not wish to lose the high-quality multi-colored surfaces generated with GraphiteLifeExplorer once in Maya, Blender or UCSF Chimera, or the user wishes to keep a geometric object like a tube representing an unstructured linker for which no atomic structure does exist.

## Implementation Details

### Graphite

Graphite (http://alice.loria.fr/index.php/software.html) is the program hosting the GraphiteLifeExplorer plugin. It is a research application, written in C++ and Python, developed with the goal to simplify the development of new geometry processing algorithms (e.g., for computing surface parameterizations or for re-meshing surfaces). Since the techniques involved are generic they can be used for other purposes such as the development of our GraphiteLifeExplorer plugin.

Graphite provides compile-time introspection that is used to automatically generate user-interfaces for C++ functions. That way, the selected functions in a plugin automatically become commands in the graphics interface (available in menus). Graphite offers similar helpers for implementing tools relying on mouse events.

A particularly useful feature of Graphite is its ability to dynamically (at run-time) attach any kind of attribute to elements of a geometric object. For example, we can load a PDB file as a set of points in space, and attach the radius and the name of each atom as attributes to the points, without having to declare new C++ data-structures.

All these features make Graphite a very developer-friendly environment for implementing innovative geometric algorithms. Furthermore, the graphical user interface (GUI) is sufficiently simple to be accessible to the casual user.

### Implementation of the DNA Tool

We choose to use a smooth curve *C* to model DNA: at any point on the curve, the tangent vector must be well defined. So must be the arc-length from the start of the curve to that point.

We use two point samplings of *C*. An *adaptive sampling* of *C* consists of a sequence of point on *C* that forms a linear approximation of the curve by straight line-segments such that the angle between two consecutive segments is below some threshold. This adaptive sampling is used to display a visually smooth curve with a minimal number of line-segments. It is used solely for visualizing the helical axis (or DNA itself when it is far from the viewpoint).

We also use a *uniform sampling S_u_* of *C*, in which all sub-curves between consecutive sample points have the same arc-length. We use a spacing close to 3.4 Å to obtain a uniform sampling *S_u_* in which the samples can serve as anchor points for base pairs of DNA.

#### A rigidly moving frame

**Figure 11:**
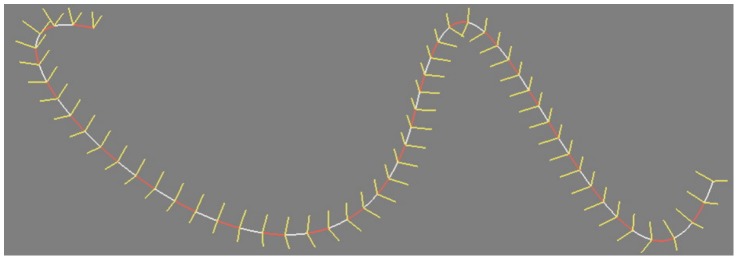
A rigidly moving frame. A uniform, rotation minimizing frame sequence along a cubic Bézier curve. The normal and binormal vectors are shown yellow.

From the above, we have obtained a uniform sampling *S_u_* of the curve together with the tangent vector at every sample point. In order to coherently orient the base pairs along the curve, we need to augment this data with a normal vector, so as to obtain an orthonormal frame at each sample point. Many simple methods to do so result in a sequence of frames exhibiting discontinuities or strong torsion (e.g., the Frenet frame [15]). But ideally, we would like the frames in the sequence to transform as rigidly as possible from one to the next (i.e. without discontinuities and minimizing torsion). Indeed, such a *rotation minimizing* frame sequence allows to model a DNA strand at rest, without torsion. Obtaining a rotation minimizing frame sequence is not an easy task, but Wang *et al.* [16] show how one can obtain an extremely good approximation at a very low computational cost. After computing a normal vector at each sample point of *S_u_* in this way, we store the sequence of triplets *F_u_ = { f_i_ = (o_i_, t_i_, n_i_), i ∈ [1,N*] } where *t_i_* is the tangent vector and *n_i_* is a normal vector to the curve *C* at the sample point *o_i_* (Figure 11). The uniform sampling *S_u_* and the frame sequence *F_u_* are recomputed from scratch whenever the user modifies the control points of the curves. Our optimized implementation is sufficiently fast for interactive modeling.

#### The DNA strand model

GraphiteLifeExplorer does not support a specific sequence of nucleotides yet, and thus assumes a generic one: (ACTG)*. We keep in RAM an atomic model centered at the origin for each four possible base pairs AT, TA, CG and GC. In order to model or draw the full DNA strand, we instantiate the atomic (3D) model at each sampling point *o_i_* of *S_u_* in the local frame *f_i_*. To reproduce the DNA helix, the model is rotated by ∼ i*2*π/10.4 rad around the tangent *t_i_*. In this way, memory cost is kept to a minimum and DNA strands of several millions base pairs easily fit in an average PC RAM.

When exporting the DNA strand to a PDB file, the instantiation of a base pair corresponds to the writing of the PDB records for each atom in the base pair, with proper position in space.

**Figure 12:**
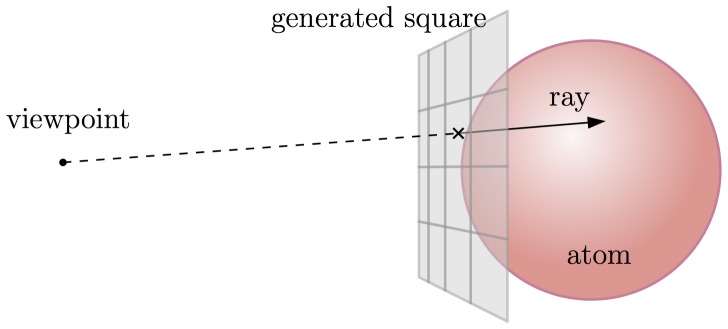
Casting a ray towards an atom.

When drawing the DNA strand to the screen, the instantiation is performed by the GPU (graphics processing unit), driven by the OpenGL API: For each base pair *i*, the local-to-global coordinate transformation for frame *f_i_* is loaded into GPU memory. Then, the centers, colors and radii of the base pair atoms are streamed to the GPU. Each atom center is transformed (by a *geometry shader)* into a square, tangent to the atom ball and facing the viewpoint. Further, a *pixel shader* is set to cast a ray from each rasterized pixel away from the viewpoint towards the atom ball (Figure 12). In this way we obtain the same results as a ray-traced image, but at a much lower cost, thus retaining interactivity.

#### Twisting strands

It is crucial for the user to be able to fine tune the position of the DNA atoms individually; at least locally when there is a strong interaction with, say, a protein residue. Allowing independent atom placement, while most powerful, would destroy the curve abstraction that we use to make DNA modeling easier. Instead, we allow the adjustment of the twist of each base pair around the curve. That is, at each frame *f_i_*, the base pair is rotated by angle ∼ i*2*π/10.4+*a_i_*, where *a_i_* is specified by the user. More precisely, *a_i_*  = 0 initially, and the user can then modify the angle of each base pair individually. The values are interpolated where they are not specifically defined: Suppose that the user has defined the values {*ai_1_, ai_2_… ai_k_*} with *i_1_< i_2_< …< i_k_*. If *i_1_*>1 we artificially set *i_0_* = 1 and *a_1_* ← *ai_1_* and similarly beyond *i_k_*. Then for all *i*, there exists *j* so that *i_j_ ≤ i<i_j+1_*, and the value *a_i_* is a linear blend of *ai_j_* and *ai_j+1_* interpolating the twisting constraints at positions *i_j_* and *i_j+1_* (Figure 5).

### Real-time Visualization

The previous section explained how the *atomic* model of a DNA strand is drawn on the screen. When the strand is very long, many base pairs are sufficiently far away from the viewpoint so as to cover only a small part of the screen. The atomic model is thus not appropriate since the atoms have a size comparable to that of a pixel, leading to objectionable flickering. For this reason, strands in the distance are drawn with simplified models: a ribbon model for moderately far strands, and a simple line for very far ones. In this way, we retain a pleasing and interactive visualization of long strands. We use a hierarchical decomposition to select a display model for each part of the strand, as detailed below.

#### A hierarchy for interactive visualization and base pair selection

We build a binary hierarchy over the sequence of frames *F_u_*. The root node consists of the entire sequence. The subsequence at one node is split in two sub-subsequences of equal length to form the two child nodes. At each node, a sphere bounding all the sampling points in the subsequence of the node is computed. We stop splitting a node when it contains about ten base pairs.

When drawing a long DNA strand, the hierarchy is traversed top-down. If the bounding sphere of the node is sufficiently far away, we use the alternative, simpler display models for that node's subsequence.

The hierarchy is also used to quickly determine which base pair the user has clicked on when he wants to twist the DNA.

#### Protein surface meshing and visualization

**Figure 13:**
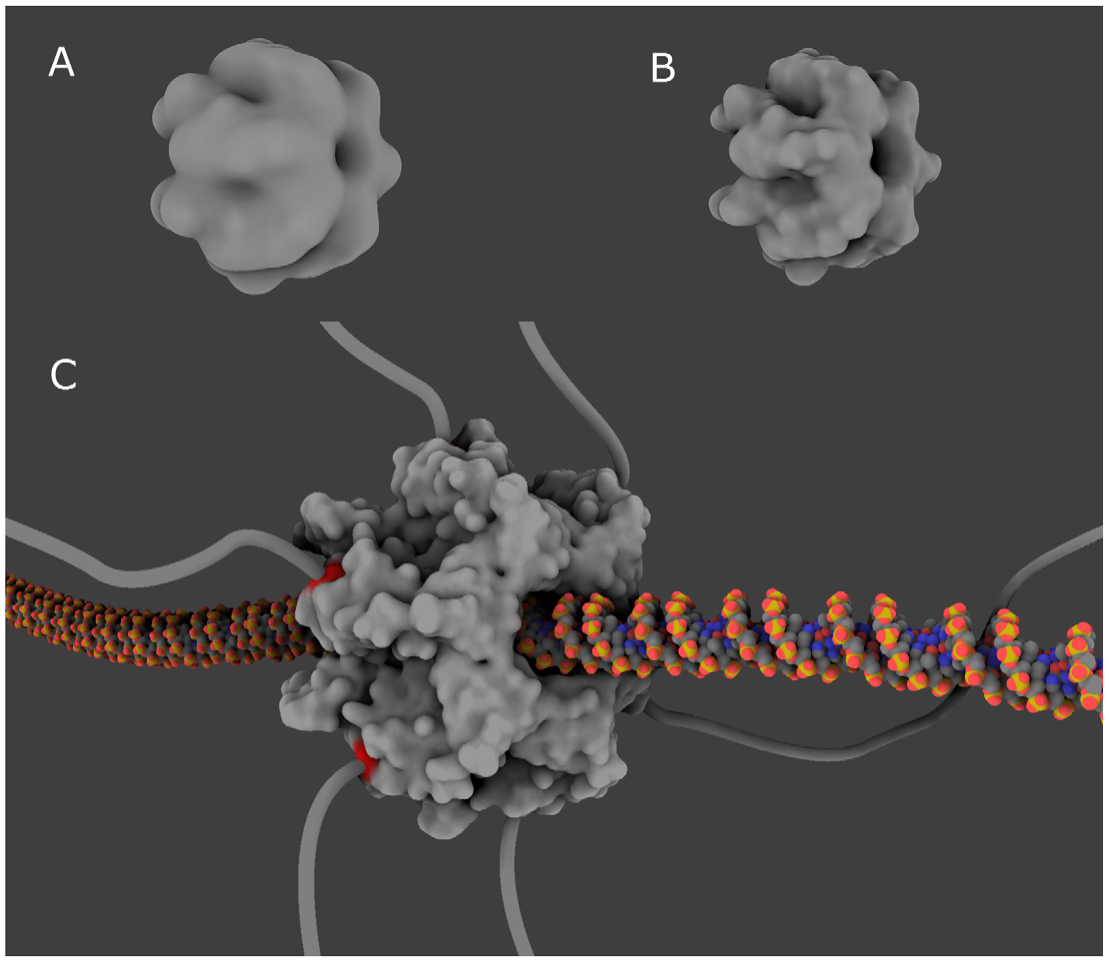
Surface meshing. The structure corresponding to the PDB code 2IUU is imported and an isocontoured surface is created showing its shape. This shape is shown at three different precision levels from coarsed-grain (A, B) to molecular skin (C). Specific residues at the surface are highlighted in red to help the connection of this hexameric protein with six linkers.

GraphiteLifeExplorer provides a tool for computing a mesh of the surface of a molecule (Figure 13). We use the ESBTL library for parsing and writing PDB files [17]. We use the surface model proposed by Grant *et al.* [18] and compute a linear approximation of it using the algorithm of Boissonnat and Oudot [19] as implemented in the CGAL library (Computational Geometry Algorithms Library http://www.cgal.org). The algorithm ensures that the mesh triangle size adapts to the local curvature of the surface, leading to high quality surface, even for low triangle budget.

More importantly, the tool allows for selecting subsets of a protein residues and coloring the parts of the protein (or DNA) surface that are closer to these residues than to others. In this way, one can visually locate selected residues on the molecule surface, in order to ease subsequent modeling.

## Conclusions

3D modeling has proved its use beyond education and illustration as it helps to build our understanding [4] by formulating hypotheses and new avenues of research [20]. With the constant flow of new information regarding the spatio-temporal organization of living systems, the development of integrative tools [21] linking architecture, genetic information and metabolism is a key step to understand how processes are performed inside the spatially structured cells. At the current stage, GraphiteLifeExplorer enables to build spatial arrangements of proteins and nucleic acids representing cellular processing, while looking for a complete bacterium in the future. To our knowledge, GraphiteLifeExplorer is the first interactive tool with the capability to freely model the shape of DNA strands, and is far easier to use than previous approaches using generic 3D modeling software.

In addition to providing new integrative tools, the lack of common biological scene repositories needs to be addressed. The use of a standard format for macromolecular assemblies would favor its adoption in the scientific community. Such standard would drive future developments of software with a high potential to foster research and discovery processes in biology [22]–[23]. The establishment of the “Working Group on Theoretical Structural Models Validation" [24] is a step in this direction.

## Availability

GraphiteLifeExplorer supports Windows Seven, Linux and Mac OS X (Lion). The pre-compiled program is freely available at http://www.loria.fr/~shornus/FFG/gle.html. The source code can be downloaded from http://gforge.inria.fr/frs/?group_id=1465. Tutorials are available from http://www.lifeexplorer.eu/3d-models/tutorials.
